# Identification of an anti-inflammatory action of exosome release in P2Y_4_ loss-mediated cardioprotection

**DOI:** 10.3389/fphar.2025.1664015

**Published:** 2025-10-14

**Authors:** Esteban Diaz Villamil, Paul Rouvier, Michael Horckmans, Lucas De Roeck, Erika Hendrickx, Louise Conrard, Didier Communi

**Affiliations:** ^1^ Institute of Interdisciplinary Research, IRIBHM, Free University of Brussels, Brussels, Belgium; ^2^ Center for Microscopy and Molecular Imaging (CMMI), Université Libre de Bruxelles, Gosselies, Belgium

**Keywords:** exosome, P2Y receptor, cardioprotection, PD-L1, ischemia

## Abstract

**Introduction:**

Exosomes are major actors in the progression of cardiovascular diseases and potential associated-treatments. We showed previously that inactivation of the mouse P2Y_4_ nucleotide receptor induces a protection against myocardial infarction in the left anterior descending artery ligation model, characterized by smaller infarcts and reduced cardiac fibrosis and inflammation, compared to wild-type mice. This cardioprotection was associated with adiponectin and PD-L1 overexpression, regulatory leukocyte increase, and adipocyte beiging in the pericardial adipose tissue of P2Y_4_-null mice. We investigated here the contribution of exosome release in the cardioprotection observed in ischemic P2Y_4_-null mice.

**Methods and results:**

Interestingly the reduction of cardiac fibrosis and T cell infiltration observed in P2Y_4_-null compared to wild-type ischemic heart was abolished after intraperitoneal injection of the exosome inhibitor GW4869 during myocardial infarction onset, as previously observed using an anti-PD-L1 blocking antibody. Additionally, GW4869 injection totally inhibited the increase in plasma PD-L1 level observed in P2Y_4_-null ischemic mice, as well as the higher T cell apoptosis in their pericardial adipose tissue, compared to wild-type mice. We observed increased expression of CDH13/T-cadherin, essential for adiponectin-driven exosome biogenesis, in P2Y_4_-null pericardial adipose tissue. Plasma exosomes were isolated from wild-type and P2Y_4_-null ischemic mice and characterized using nanoparticle tracking analysis and transmission electron microscopy experiments, as well as Western blot analysis of CD63 exosome marker and adiponectin expression. Our data support an increase in exosomes from adipocyte origin in the plasma of P2Y_4_-null ischemic mice. Flow cytometry experiments showed that P2Y_4_-null ischemic mice displayed an increased level of PD-L1^+^ plasma exosomes compared to wild-type ischemic mice. We finally demonstrated the capacity of total plasma exosomes from P2Y_4_-null ischemic mice to polarize macrophages into the anti-inflammatory M2c phenotype *in vitro*. M2c macrophages can inhibit T cell activation through PD-L1 regulation and play a central role in the resolution of cardiac inflammation to promote cardiac repair.

**Discussion:**

These data support the role of the release of anti-inflammatory exosomes, and more particularly the exosomal form of PD-L1 and adiponectin, in P2Y_4_ loss-mediated cardioprotection. The study of regulators of cardioprotective exosomes could lead to the development of novel anti-inflammatory therapies to improve myocardial infarction outcome.

## Introduction

The nano-sized extracellular vesicles called exosomes are now considered as a rising star in failing hearts by regulating cardiac function in health and disease (Xu et al., 2017). In contrast to mesenchymal stem cell-based therapies, exosomes display no apparent adverse effect based on their stability, biocompatibility, low toxicity and low immunogenicity (Xu et al., 2017). Interestingly, exosomes from mouse adipose-derived stem cells (ADSCs) have been shown to reduce coronary oxidative stress-induced cardiomyocyte apoptosis after ischemia/reperfusion injury (Liu et al., 2019; Lai et al., 2020). Injection of ADSC-derived exosomes reduced ischemia-induced injury after myocardial infarction in rats (Cui et al., 2017). ADSC-derived exosomes were also shown to promote cardiac angiogenesis after a myocardial infarction (Wang et al., 2023). Transplanted mesenchymal stem cells can also exert a cardioprotective action through exosomes, that mainly depends on circulating adiponectin (Nakamura et al., 2020).

While pro-inflammatory exosomes can participate to the progression of cardiovascular disease, exosomes can also be designated as carriers of cardioprotective proteins and miRNAs enhancing the survival of recipient cells during ischemia and displaying a therapeutic potential (Rosand and Hoydal, 2021). Plasma exosomes can protect the myocardium from ischemia-reperfusion injury by acting on cardiomyocyte survival through specific miRNAs or heat shock proteins (Minghua et al., 2018; Vicencio et al., 2015). The study of the function and cargo of pro- and anti-inflammatory exosome subsets is determinant to understand the cellular crosstalk that initiates adaptive processes such as cardiac repair. Exosomes originating from damaged cardiac cells are potential biomarkers to evaluate severity and stage of a heart attack (Yuan et al., 2016; Liu et al., 2021; Wang M. et al., 2025). Their study will also better define the future therapeutic use of anti-inflammatory exosomes in cardiovascular disease.

A better understanding of the molecular mechanisms and cellular mediators that control tissue plasticity and tissue-derived exosome release is essential to act on the main actors in the chosen treatment after myocardial infarction (MI). Exosomes secreted from cardiac cells such as cardiomyocytes and cardiac endothelial cells, adipocytes and stem cells can participate to post-ischemic response and cardiac remodeling (Zhang et al., 2023). MI induces the release of a complex mixture of pro- and anti-inflammatory circulating exosomes from various cell/tissue origins. These exosome subsets are characterized by multiple signatures of specific surface markers, miRNAs, soluble proteins and lipids. Among the bioactive molecules transported in circulating exosomes, miRNAs play determinant roles in exosome functions and have been associated with cardiac fibrosis, atrial function and cardiac output regulation (Zhang et al., 2023). In obese individuals, adipose tissue-derived exosomes (AT-exosomes) contribute to the development of insulin resistance and secretion of pro-inflammatory cytokines (Zhang et al., 2016). In post-infarct conditions, nano-sized vesicles are released into the bloodstream by circulating cells such as leukocytes and platelets, and also by inflamed adipose tissues and the ischemic heart (Yuan et al., 2016). Pro-inflammatory exosomes are known to influence macrophage polarization by shifting the balance towards M1 macrophages and by interacting with fibroblasts to induce excessive fibrosis and adverse cardiac remodeling worsening heart injury (Rosand and Hoydal, 2021; Bellin et al., 2019). The identification of cardioprotective exosomes directly contributing to the resolution of cardiac inflammation represents a major interest in treatments against MI.

We previously described the role of mouse nucleotide P2Y_4_ receptor in protection against MI (Horckmans et al., 2015). The P2Y_4_ receptor, is a UTP G-protein coupled receptor in human (Communi et al., 1995), and is activated by both ATP and UTP in mouse (Communi and Boeynaems, 1997). We showed that mouse P2Y_4_ knock-out (KO) mice displayed reduced infarct size and absence of left ventricular hypertrophy, in the left anterior descending coronary artery ligation (LAD ligation) model (Horckmans et al., 2015). Mouse P2Y_4_ receptor was detected in different adipose tissues, predominantly in pericardial adipose tissue (PAT), both in cardiac adipocytes and cardiac ADSCs (Lemaire et al., 2017). P2Y_4_ inactivation induced increased adiponectin production and adipocyte beiging in pericardial adipose tissue (PAT) (Lemaire et al., 2017). The interaction between adiponectin and CDH13 enhances exosome biogenesis and secretion by many cell types including endothelial cells, pericytes and mesenchymal stem cells (De et al., 2010; Parker-Duffen et al., 2013; Matsuda et al., 2015).

Besides their overexpression of adiponectin, we demonstrated that P2Y_4_ KO mice displayed also increased expression of PD-L1 (Programmed Death-Ligand 1) immune checkpoint in their PAT after LAD ligation, compared to WT mice (Horckmans et al., 2022a). Interestingly, the reduction of cardiac inflammation and fibrosis caused by the loss of mouse P2Y_4_ receptor was abolished by intraperitoneal injection of an anti-PD-L1 blocking antibody (Horckmans et al., 2022a). We also showed that adiponectin and PD-L1 overexpression in P2Y_4_ KO mice was associated with an increase of regulatory T cells (Tregs) and M2c macrophages in their ischemic PAT, compared to WT mice (Horckmans et al., 2022a). PD-L1 is also a recognized marker of cardioprotective beige adipocytes (Ingram et al., 2017), that are increased in P2Y_4_ KO mice (Horckmans et al., 2022a). The role of PD-L1 immune checkpoint in cardiovascular disease has been poorly investigated. In an autoimmune myocarditis model, PD-L1 protects the heart from excessive inflammation and damage (Lucas et al., 2008). Adipose-derived stem cells overexpressing PD-L1 can ameliorate cardiac function and attenuate infarct size by upregulating regulatory T cells (Tregs) in acute MI rat model (Lin et al., 2024).

We investigated here the potential production of anti-inflammatory and cardioprotective exosomes release, and more particularly PD-L1^+^ exosomes in P2Y_4_ KO mice. The exosomal form of PD-L1 is described as an important actor in tumor immunotherapy (Poggio et al., 2019). We evaluated here the possible contribution of plasma anti-inflammatory exosome release to post-MI cardiac fibrosis and inflammation using exosome inhibitor injection in P2Y_4_ KO mice. Their plasma exosomes were characterized using high-sensitivity flow cytometry, Western blotting, Nanoparticle Tracking Analysis (NTA) and Transmission Electron Microscopy (TEM), as well as *in vitro* leukocyte polarization experiments.

## Materials and methods

### Animals

C57BL/6J P2Y_4_ KO mice were generated in our laboratory (Robaye et al., 2003). Adiponectin KO mice named B6; 129-Adipo^tm1Chan^/J were purchased at JAX, The Jackson Laboratory (Bar Harbor, ME, United States). C57BL/6J P2Y_4_/adiponectin double KO mice were generated in our laboratory (Horckmans et al., 2022a). 10- to 14-week-old male and female wild type (WT), P2Y_4_ KO, Adiponectin KO and P2Y_4_/adiponectin double KO mice were used randomly.

### Ischemia *in vivo* experiments: LAD ligation

MI was induced by permanent ligation of the left anterior descending coronary artery, as previously described (Horckmans et al., 2015). Mice were anesthetized with midazolam (5 mg/kg), medetomidine hydrochloride (0.5 mg/kg) and fentanyl (0.05 mg/kg), intubated, and mechanically ventilated with a MiniVent mouse ventilator (Harvard Apparatus, Holliston, MA, United States). Optical magnification loop was used for better visualization of the operation field. A left thoracotomy was performed in the fourth left intercostal space, and the pericardium was carefully incised to maintain the integrity of the PAT. Once the heart was exposed, MI was induced by the permanent ligation of the left anterior descending artery (LAD) proximal to its bifurcation from the main stem. Successful performance of coronary occlusion was confirmed by blanching of the myocardium distal to the coronary ligation. The thoracic incision was then closed with a 5–0 silk suture (Covidien, Dublin, Ireland) at the muscle tissue and a 7–0 silk suture (Covidien, Dublin, Ireland) at the skin. After surgery, naloxone hydrochloride (1.2 mg/kg), flumazenil (0.5 mg/kg) and atipamezole hydrochloride (2.5 mg/kg) which are the respective antagonists of the used anesthetics were administered to reverse the effect of anesthesia and for an immediate and secure recovery of the mice. Postoperative analgesia (buprenorphine, 0.1 mg/kg) was given for the first 12 h to support animal wellness after surgery.

### Injection of GW4869 exosome inhibitor in ischemic mice

To evaluate the importance of exosomes in our model, we analyzed the *in vivo* effect of GW4869 exosome release inhibitor (Sigma-Aldrich, St. Louis, MO, United States) 24 h or 7 days post-MI. For experiments realized 24 h post-MI, the GW4869 exosome inhibitor was intraperitoneally injected (2.5 μg/g) 1 h before LAD ligation. For experiments realized 7 days post-MI the GW4869 exosome inhibitor was intraperitoneally injected (2.5 μg/g) 1 h before LAD ligation and 1, 3 and 5 days after LAD ligation.

### Quantification of cardiac fibrosis area in ischemic hearts

Paraffin cross-sections (8 µm) of infarcted hearts were cut, fixed in Bouin’s solution (Sigma-Aldrich, St. Louis, MO, United States) and stained with Masson’s trichrome (Sigma-Aldrich, St. Louis, MO, United States), following manufacturer recommendations. Images of whole hearts were acquired with NanoZoomer-SQ (Hamamatsu Photonics, Hamamatsu, Japan) at 0.23 μm/pixel. Fibrosis was quantified as the relative area of the blue staining (collagen) compared to the left ventricle surface on at least four sections at different levels per ischemic heart, using ImageJ software.

### Immunofluorescence experiments

Hearts were harvested, weighted and immediately frozen in Tissue-Tek OCT compound (VWR Scientific). Frozen heart sections were cut at 8 µm thickness and fixed with methanol. Heart sections were stained with an antibody against CD3 (clone 17A2, 1:500, BD Biosciences, Franklin Lakes, NJ, United States). Images of immunostaining were acquired at room temperature in FluorSave reagent (Calbiochem) using an Axio Observer Z1 wide-field microscope equipped with a high-resolution charge-coupled device mono camera (Zeiss Axiocam 702) using a EC Plan NeoFluar x10/0.3 dry objective (Zeiss), and AxioVision 4.6.3 software (Zeiss). T cell density was quantified using ImageJ software by examining 10 fields per section at ×100 magnification, in a blinded fashion. Sections were counterstained with Hoechst to visualize the entire population of cell nuclei within each myocardial section. CD3 counts were performed by examining at least three sections per heart in the border zone of myocardial infarct, with comparable nuclear detection, For adiponectin and CDH13 staining, frozen sections (5 µm) of mouse PAT were stained with antibodies against adiponectin (ab181281, abcam, Cambridge, UK) and CDH13 (ab317440, abcam, Cambridge, UK). Sections were counterstained with Hoechst. For all histological examinations, at least three sections per mouse were analyzed.

### Study of T cell apoptosis in the PAT

PAT of control mice or mice subjected to LAD (7 days post-MI) and injected or not with the GW4869 exosome inhibitor (2.5 μg/g), was freshly harvested after perfusion with PBS to remove peripheral cells. PAT was then finely minced and digested in collagenase A solution (2.5 g/L collagenase A (Roche, Mannheim, Germany)) at 37 °C for 45 min. The digested tissue was centrifuged at 500 g for 5 min to separate the stromal vascular fraction form mature adipocytes. The supernatant containing mature adipocytes was discarded. Red blood cell lysis was performed by osmotic shock with ACK (ammonium-chloride-potassium) lysis buffer. The stromal vascular fraction was resuspended in PBS supplemented with 3% FBS and CD16/CD32 Fc‐block (clone 2.4G2, BD Pharmingen, BD Biosciences, Erembodegem, Belgium) and stained with a mix of fluorochrome‐conjugated antibodies for 1 h on ice. Antibodies used were CD45 (clone 30F11), CD3 (clone 17A2) and annexin V (all from BioLegend). Data were acquired on a BD LSRFortessa cell analyzer running BD FACSDiva software (BD Biosciences, Franklin Lakes, NJ, United States), and analysis was performed with FlowJo software (Ashland, OR, United States).

### ELISA experiments

Plasma PD-L1 levels were measured in the plasma of WT and P2Y_4_ KO sham or ischemic mice using the mouse PD-L1 ELISA DuoSet kit (R&D systems, Minneapolis, MN, United States) following manufacturer recommendations.

### Exosome isolation and exosomal protein quantification

Exosomes were extracted from the plasma of WT and P2Y_4_ KO sham and ischemic mice using the Exo-spin Exosome Purification Kit (Cell Guidance Systems, Cambridge, UK) and following manufacturer recommendations. Total exosomal proteins were quantified using the Pierce BCA Protein Assay Kit (Thermo Scientific, Rockford, IL, United States) following manufacturer recommendations.

### Western blotting experiments

Exosomes isolated from mouse plasma were lysed for 10 min on ice in 200 μL RIPA lysis buffer (50 mM Tris, 150 mM NaCl, 1% Triton X-100, 0.5% sodium deoxycholate, 0.1% SDS, pH 8.0) containing protease inhibitor cocktail (Roche Diagnostics, Penzberg, Germany). Lysates were mixed 5:1 with 5x Laemmli buffer (0.625 M Tris–HCl, 10% SDS (w/v), 50% glycerine, under reducing conditions with 25% DTT, bromophenol blue, pH 6.8) and incubated for 5 min at 70 C. The membrane was blocked for 1 h at room temperature with blocking buffer consisting of 5% (w/v) non-fat dry milk in TBST (50 mM Tris-buffered saline, 150 mM NaCl, 0.05% Tween 20, pH 7.5). Adiponectin and CD63 were detected with anti-adiponectin and anti-CD63 antibodies (R&D Systems) followed by anti-goat-HRP (Cell Signalling, Danvers, MA, United States) as secondary antibody. Incubation with antibodies was carried out in 0.5% (w/v) non-fat dry milk in TBST overnight (primary antibody) or for 1 h (secondary antibody) at room temperature. Visualization was accomplished using Chemiluminescence Reagent Plus (Roche Diagnostics, Diegem, Belgium) and X-ray films (Hyperfilms ECL, GE Healthcare, Munich, Germany).

### Particle analysis by flow cytometry

The presence of specific cell markers on plasma exosomes was assessed by flow cytometry. Plasma exosomes (45 µL) isolated using the Exo-spin Exosome Purification Kit were incubated with antibodies against CD63 (clone NVG-2, 2/500), PD-L1 (clone 10G.9G2, 2/500), CD31 (clone MEC 13.3, 2/250) and CD41 (clone MWReg30, 2/500). To ensure accurate compensation, single-stained controls were prepared for each fluorophore used, along with an unstained control exosome suspension and were used to calculate a compensation matrix, to correct for spectral overlap between fluorochromes. Size gating was established using calibration beads (Flow Cytometry Sub-micron Particle Size Reference kit, Invitrogen™) as references and exosomes were identified by gating on CD63 positive events. Data were acquired on a BD LSRFortessa cell analyzer running BD FACSDiva software (BD Biosciences, Franklin Lakes, NJ, United States), and analyzed with FlowJo software (Ashland, OR, United States).

### Nanoparticle tracking analysis of plasma exosomes

The size distribution, volume and concentration of plasmatic particles isolated using the Exo-spin Exosome Purification Kit were determined using a Nanoparticle Tracking Analyzer (Zetaview, Particle Metrix, Germany) equipped with a 488 nm laser. For each measurement, 11 cell positions were scanned and 60 frames by position were captured. The data were processed using the in-build ZetaView Software (8.05.12 SP1), with analysis parameters set as: maximum size: 1,000, minimum size: 10, and minimum brightness: 20.

### Cryo-transmission electron microscopy (TEM) analysis of plasma exosomes

For Cryo-TEM analysis, QUANTIFOIL^®^ R1.2/1.3 200 mesh Cu grids were glow-discharged using an ELMO glow discharge system (Cordouan Technologies) at a vacuum of 2.1 × 10^−1^ mbar and a voltage of 1.7 V for 35s. Grids were then transferred to a Vitrobot Mark IV (Thermo Fisher Scientific) for a plunge-freezing procedure. A double application of samples was used: 3 µL of plasma particle suspension obtained using Exo-spin was applied to the grid, followed by a blotting (blot force = 2, blot time = 3s, wait time = 5s). Immediately after the first blot, a second 3 µL aliquot was applied, followed by a final blot (blot force = 2, blot time = 3s, wait time = 0s). Vitrified grids were transferred into a Talos transmission electron microscope (Thermo Fisher Scientific) and observed under cryogenic conditions at an accelerating voltage of 200 kV. Images were acquired using EPU software (Thermo Fisher Scientific) and a Falcon III EC camera at a magnification of ×73,000 corresponding to a calibrated pixel size of 0.14 nm/pixel. The total electron dose was approximately 21 e^−^/Å^2^.

### Effect of exosomes on the polarization of isolated bone marrow-derived macrophages

Exosomes were extracted from the plasma of WT, P2Y_4_ KO, adiponectin KO and P2Y_4_/adiponectin double KO sham and ischemic mice (24 h or 7 days post-MI) using the Exo-spin kit (Cell Guidance Systems, Cambridge, UK). Bone marrow-derived macrophages (BMMs) were isolated from bone marrow flushed from femurs of mice. The bone marrow was freshly harvested, suspended in PBS and centrifuged at 300 g for 5 min. Red blood cell lysis was performed by osmotic shock with ACK (ammonium-chloride-potassium) lysis buffer. Cells were then plated and cultured in RPMI 1640 medium supplemented with 10% fetal bovine serum, 1% penicillin-streptomycin and 20% L929 cell-conditioned medium (as a source for M-CSF) for 7 days, with a complete change of medium every 2 days. Cultures were incubated at 37 C, in a humidified 95% air-5% CO_2_ atmosphere. To assess the role of exosomes on macrophage polarization, they were incubated with plasma-derived exosomes during 48 h before flow cytometry experiments using antibodies against CD45 (clone 30F11), F4/80 (clone BM8) and CD206 (all from BioLegend) and MerTK (AF591, R&D, Abingdon, UK). Data were acquired on a BD LSRFortessa cell analyzer running BD FACSDiva software (BD Biosciences, Franklin Lakes, NJ, United States), and analysis was performed with FlowJo software (Ashland, OR, United States).

### Statistics

All the data obtained are expressed as mean ± SEM, and statistical analysis was performed with GraphPad Prism software (version 6; GraphPad Software, San Diego, CA, United States). Normality and homoscedasticity of data distribution were assessed using respectively the Shapiro–Wilk test and Levene’s test. Endpoint comparisons between two groups were performed using unpaired two-tailed Student’s t-test. For comparisons involving multiple groups, two-way or three-ways ANOVA was used and a Bonferroni *post hoc* evaluation was performed. A two-tailed P < 0.05 was considered as significant.

## Results

### Involvement of exosome release in the reduction of cardiac fibrosis observed in P2Y_4_ KO compared to WT ischemic hearts

**FIGURE 1:**
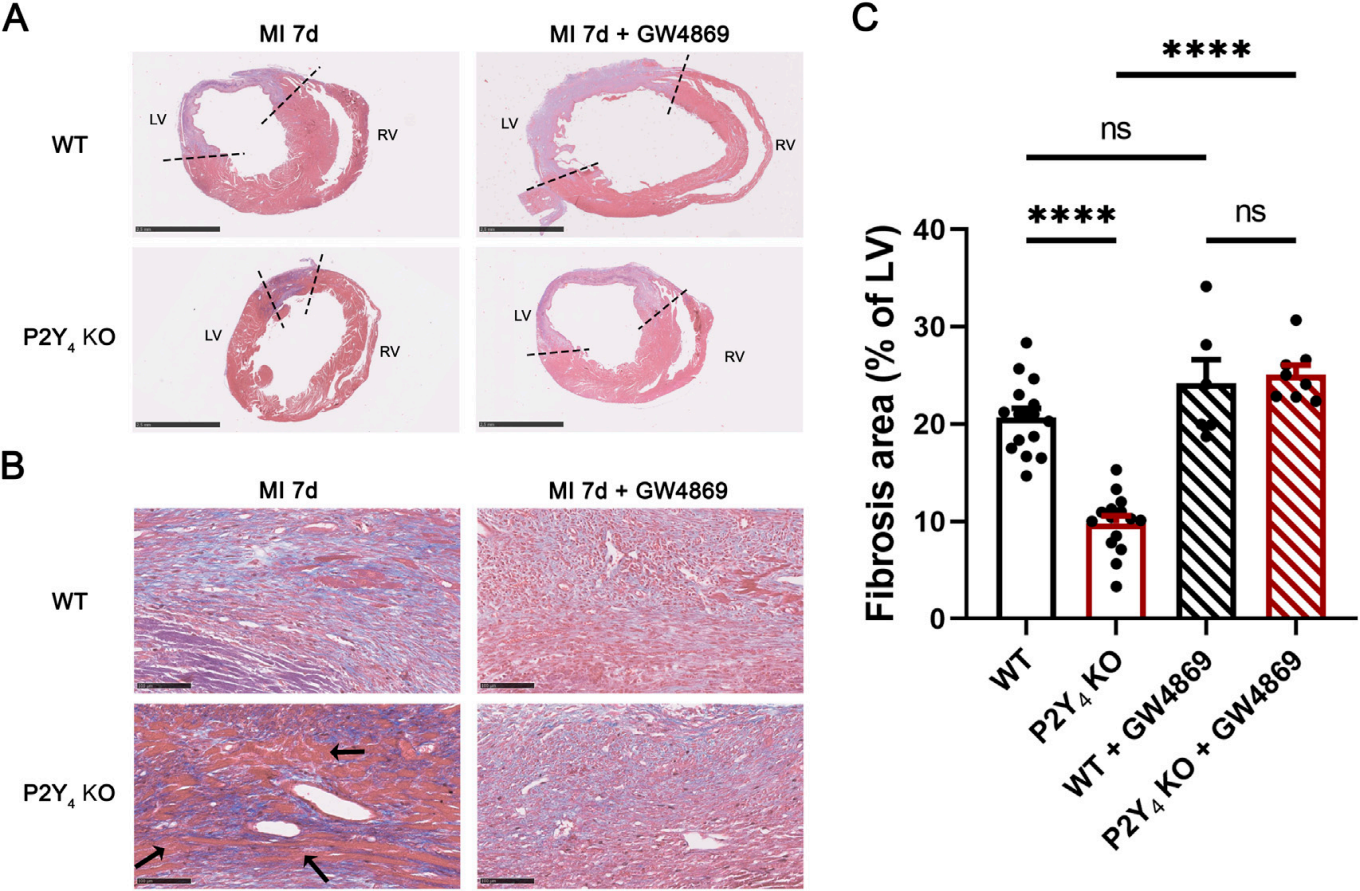
GW4869 exosome inhibitor abolishes the reduction of cardiac fibrosis observed in ischemic P2Y_4_ KO compared to WT hearts. **(A)** Representative Masson’s trichrome staining of cardiac fibrosis (collagen in blue; fibrotic infarct area between dotted lines) in WT and P2Y_4_ KO mice, 7 days post-MI, injected (MI 7d+GW4869) or not (MI 7d) with the GW4869 exosome inhibitor (2.5 μg/g) 1 h before and 1, 3 and 5 days after LAD ligation (scale bar represents 2.5 mm). **(B)** Representative Masson’s trichrome staining of cardiac fibrosis (collagen in blue) at × 20 magnification. Non-fibrotic regions present in P2Y_4_ KO heart are indicated by black arrows (scale bar represents 100 μm). **(C)** Quantification of fibrosis area normalized to total left ventricle (LV) area in WT and P2Y_4_ KO ischemic hearts, 7 days post-MI after intraperitoneal injection of GW4869 (MI 7days + GW4869) or PBS 5% DMSO (MI 7days). Fibrosis area was quantified as relative surface of collagen (blue staining) on 4-8 sections per ischemic heart using the color image analyzer ImageJ and expressed as percentages of total LV surface. The sex ratio was 40% male and 60% female mice in the WT MI 7d and P2Y_4_ KO MI 7days groups (n = 15). For the WT MI 7d + GW4869 (n = 6) and P2Y_4_ KO MI 7d + GW4869 (n = 8) groups, the sex ratio was 50% male and 50% female. Data were analyzed using a two-way ANOVA followed by a Bonferroni *post hoc* test. Data represent mean ± SEM. ****p < 0.0001.

To investigate the importance of exosome release in the cardioprotection observed in P2Y_4_ KO mice, LAD experiments were performed on mice injected with an inhibitor of neutral sphingomyelinase (N-SMase) blocking exosome synthesis and release, GW4869. WT and P2Y_4_ KO mice were subjected to LAD ligation and intraperitoneally injected, or not, with the GW4869 exosome inhibitor (2.5 μg/g) 1 h before LAD ligation and 1, 3 and 5 days after LAD ligation during myocardial infarction (MI) onset. We analyzed the effect of these intraperitoneal injections of GW4869 on cardiac fibrosis in infarcted hearts of WT and P2Y_4_ KO mice (Figures 1A,B). Hearts were harvested and embedded in paraffin 7 days after LAD ligation. Ischemic heart sections were stained with Masson’s trichrome to evaluate cardiac fibrosis. Fibrosis was quantified by calculating the area stained blue, expressed as a percentage of the left ventricle’s total area (Figure 1C). The reduction in the fibrosis area observed in P2Y_4_ KO ischemic hearts, compared with WT ischemic hearts (19.97% ± 1.12% vs. 9.80% ± 0.77%, p < 0.0001), was no longer observed in ischemic hearts of GW4869-injected P2Y_4_ KO mice (Figure 1C). At higher magnification, we observed a higher amount of healthy cardiac muscle fibers in the infarct zone of P2Y_4_ KO ischemic hearts, but not in GW4869-injected mice (Figure 1B). There was no significant difference in fibrosis area between GW4869-injected WT and P2Y_4_ KO ischemic hearts (24.17% ± 2.45% vs. 24.24% ± 1.19%) (Figure 1C).

### Injection of the GW4869 exosome inhibitor abolishes the reduction of T cell infiltration in ischemic P2Y_4_ KO hearts compared to ischemic WT hearts

**FIGURE 2:**
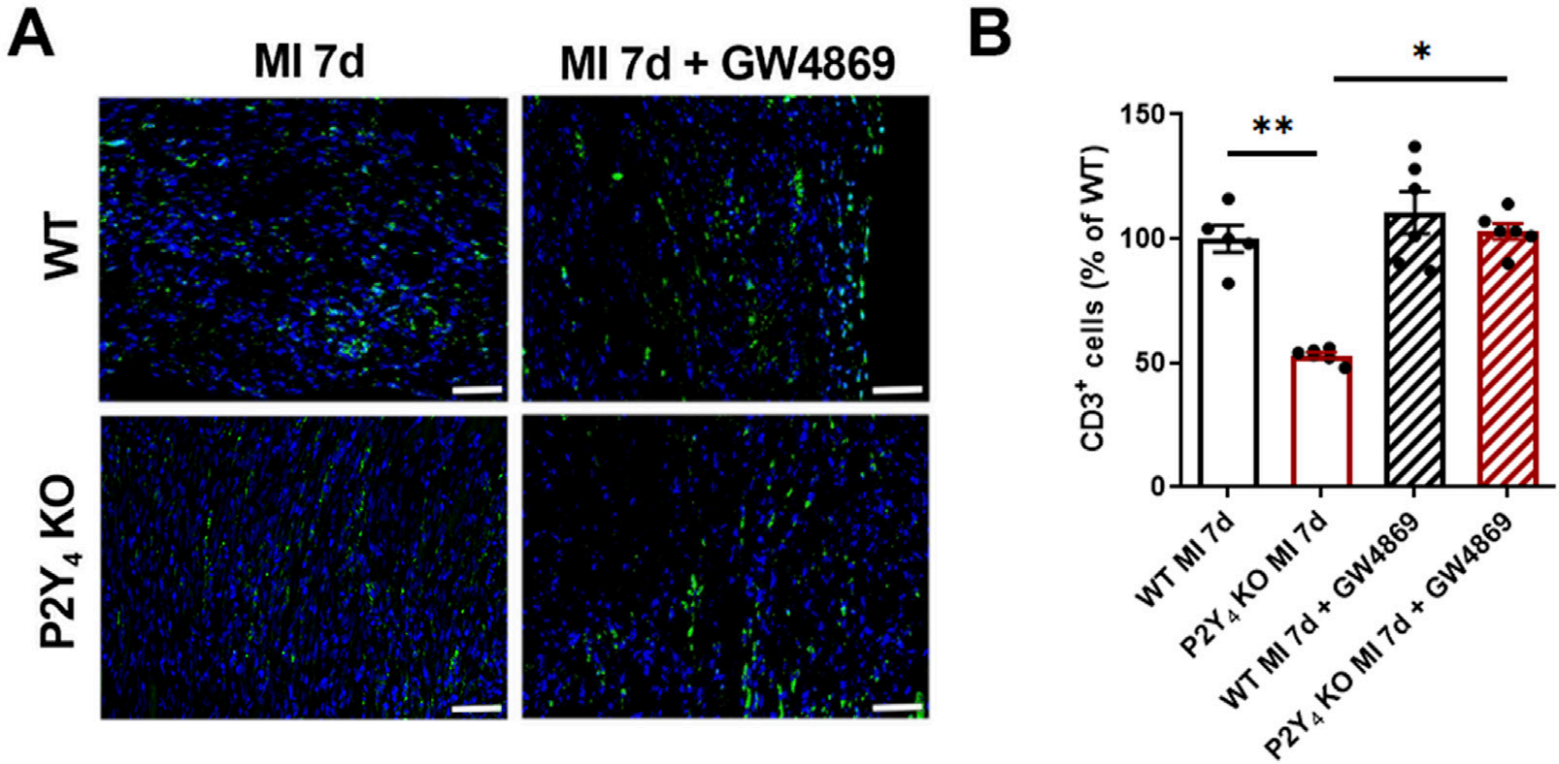
GW4869 exosome inhibitor abolishes the reduction of T cell infiltration observed in ischemic P2Y_4_ KO compared to WT heart. **(A)** Representative T cell staining (CD3) in myocardial infarct sections of WT and P2Y_4_ KO mice, 7 days after LAD ligation using anti-CD3 (green) and Hoechst (blue) (×20 magnification) (scale bar = 50 μm). Mice were intraperitoneally injected with the GW4869 exosome inhibitor (2.5 μg/g) (MI 7d + GW4869) or not (MI 7d), 1 h before and 1, 3 and 5 days after LAD ligation. **(B)** CD3^+^ cell quantification in representative counting surface/field (0.1 mm^2^) of myocardial infarct sections, 7 days after LAD ligation. Data are expressed as percentages compared to T cell number in WT ischemic hearts and obtained using ImageJ software by examining 30 fields per heart at × 100 magnification. The sex ratio was 50% male and 50% female in each group (n = 6), except for the WT MI 7d group (n = 5), which included 40% males and 60% females. Data were analyzed using a two-way ANOVA followed by a Bonferroni *post hoc* test. Data represent mean ± SEM. *p < 0.05; **p < 0.01.

We investigated cardiac inflammation by evaluating CD3^+^ T cell infiltration in the hearts of WT and P2Y_4_ KO ischemic mice 7 days post-MI. Mice were intraperitoneally injected or not, with the GW4869 exosome inhibitor (2.5 μg/g) 1 h before and 1, 3 and 5 days after LAD ligation. T cell quantification was realized in 30 fields per ischemic heart in the border zone of the infarct using ImageJ software. As previously described, we observed a significant decrease of CD3^+^ cells in the hearts of P2Y_4_ KO compared to WT ischemic mice (Horckmans et al., 2022a). Interestingly, reduced T cell infiltration was no longer observed in P2Y_4_ KO ischemic mice after injection with the GW4869 exosome inhibitor (Figures 2A,B).

### Injection of the GW4869 exosome inhibitor inhibits increased T cell apoptosis observed in the pericardial adipose tissue of P2Y_4_ KO ischemic mice

The PAT displays close interactions and shared circulation with the heart and coordinates immune cell activation within fat-associated lymphoid clusters (FALCs). We have previously identified a reduction of T lymphocytes both in the PAT and heart of P2Y_4_ KO compared with WT ischemic mice (Lucas et al., 2008). PAT could be a possible local source of exosomes that could regulate cardiac inflammation in P2Y_4_ KO ischemic mice. The interaction between adiponectin, a cardioprotective adipokine upregulated in P2Y_4_ KO mice (Horckmans et al., 2022a), and CDH13 is determinant in exosome formation and release (Obata et al., 2018). Adiponectin, is known to accumulate in tissues such as heart, vascular endothelium, and skeletal muscles through its interaction with T-cadherin, also named CDH13 (De et al., 2010; Parker-Duffen et al., 2013; Matsuda et al., 2015).

**FIGURE 3:**
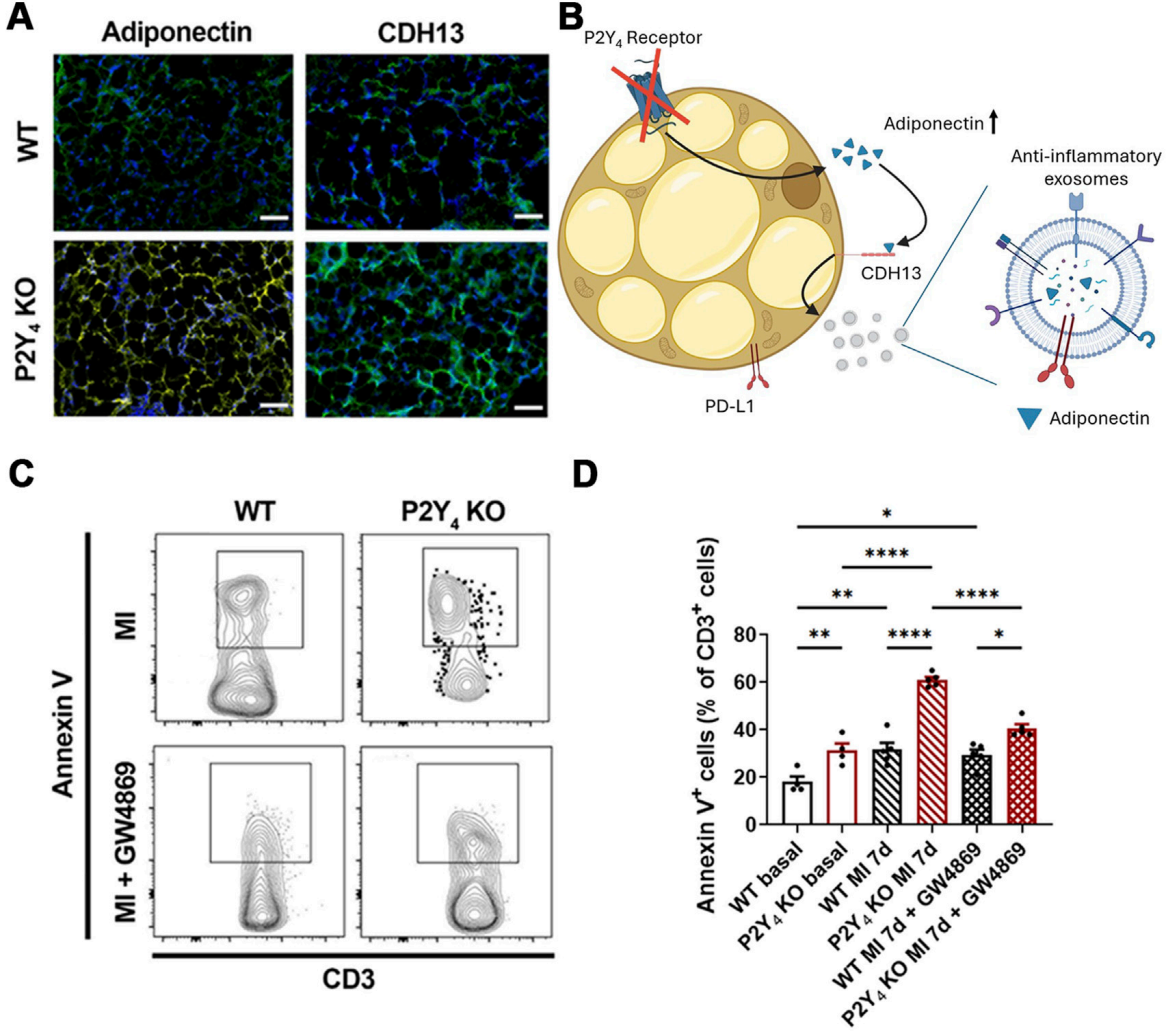
Loss of P2Y_4_ increases adiponectin and CDH13 expression and apoptosis of CD3^+^ cells in PAT. **(A)** Representative adiponectin (yellow) and CDH13 (green) immunofluorescence staining in PAT sections of WT and P2Y_4_ KO mice, 24 h post-MI (×100 magnification) (scale bar = 50 μm). **(B)** Schematic illustration of potential adiponectin-mediated release of anti-inflammatory exosomes from beige adipocytes lacking P2Y_4_ receptor. **(C)** Representative flow cytometry dotplots for annexin V^+^ CD3^+^ lymphocytes in the PAT of WT and P2Y_4_ KO mice 7 days post-MI. Mice were intraperitoneally injected (MI + GW4869) or not (MI), with the GW4869 exosome inhibitor (2.5 μg/g) 1 h before and 1, 3 and 5 days after LAD ligation. **(D)** Flow cytometry quantification of annexin V^+^ apoptotic T cells (percentage compared to total CD3 positive cells) in PAT of WT and P2Y_4_ KO mice in basal conditions or 7 days post-MI, with or without intraperitoneal injection of the GW4869 exosome inhibitor (2.5 μg/g). The sex ratio was 50% male and 50% female in the basal condition groups (WT basal and P2Y_4_ KO basal; n = 4). For the MI condition groups (WT MI 7d, P2Y_4_ KO MI 7d, WT MI 7d + GW4869, and P2Y_4_ KO MI 7d + GW4869; n = 5), the sex ratio was 40% male and 60% female. Data were analyzed using a three-way ANOVA followed by a Bonferroni *post hoc* test. Data represent mean ± SEM. *p < 0.05; **p < 0.01; ****p < 0.0001.

Histological analysis of ischemic PAT revealed an increased staining of adiponectin and CDH13 in P2Y_4_ KO mice compared to WT mice, 7 days post-MI (Figure 3A). The connections between the ischemic heart and the PAT are determinant in the inflammatory response to MI. PD-L1 immune checkpoint protein is also reported as marker of beige adipocytes (Ingram et al., 2017). We previously demonstrated adipocyte beiging as well as PD-L1 overexpression in the PAT of P2Y_4_ KO compared to WT ischemic mice (Horckmans et al., 2022a). We also observed increased adiponectin release from cardiac adipocytes of P2Y_4_ KO mice in hypoxic conditions (Lemaire et al., 2017). As illustrated in Figure 3B, we decided to investigate further a potential adiponectin-mediated release of anti-inflammatory exosomes from beige adipocytes lacking P2Y_4_ receptor.

We demonstrated previously a reduced post-ischemic FALCs expansion and an increase in T regulatory cells in the PAT of P2Y_4_ KO mice (Horckmans et al., 2022a). More particularly, we showed an increase of T cell apoptosis in the PAT of P2Y_4_ KO ischemic mice, 7 days after MI (Horckmans et al., 2022a). We inhibited here exosome release in ischemic mice and we evaluated T cell apoptosis in the ischemic PAT. WT and P2Y_4_ KO mice were injected intraperitoneally, or not, with the GW4869 exosome inhibitor (2.5 μg/g), 1 h before LAD ligation and 1, 3 and 5 days after LAD ligation. Flow cytometry experiments were performed to quantify apoptotic T cells (annexin V^+^ CD3^+^) in the PAT of ischemic mice, 7 days post-MI. We observed an increase of T cell apoptosis in the basal PAT of P2Y_4_ KO mice compared to WT (31.3% ± 2.9% vs. 18.0% ± 2.4%, p = 0.0085) which was amplified in ischemic mice 7 days post-MI (61.0% ± 1.2% vs. 31.6% ± 2.9%, p < 0.0001) (Figures 3C,D). The increase of T cell apoptosis in the PAT of P2Y_4_ KO ischemic mice was strongly inhibited after intraperitoneal injection of the GW4869 exosome inhibitor (40.6% ± 1.7% vs. 61.0% ± 1.2%, p < 0.0001) (Figures 3C,D). Nevertheless a smaller remaining increase of T cell apoptosis between the PAT of WT and P2Y_4_ KO mice was observed after the injection of the GW4869 exosome inhibitor (40.6% ± 1.7% vs. 29.4% ± 2.3%, p = 0.0141) (Figures 3C,D).

### Characterization of plasma exosomes from P2Y_4_ KO ischemic mice

P2Y_4_ receptor loss is correlated with an overexpression of adiponectin, known to be involved in exosome production (Lemaire et al., 2017). In post-infarct conditions, inflamed adipose tissues contribute significantly to the release of nano-sized vesicles in the bloodstream (Zhang et al., 2023). P2Y_4_ expression has been detected in adipose tissues, predominantly in the PAT, in cardiac adipocytes and adipose-derived stem cells (Lemaire et al., 2017). The contribution of the PAT to systemic exosome production and release in the blood is expected to be limited but P2Y_4_ expression could be enhanced in other adipose tissues and cell types in ischemic conditions. To investigate how exosome production is regulated during ischemia in P2Y_4_ KO and WT mice, we decided to isolate and study their plasma exosomes.

**FIGURE 4:**
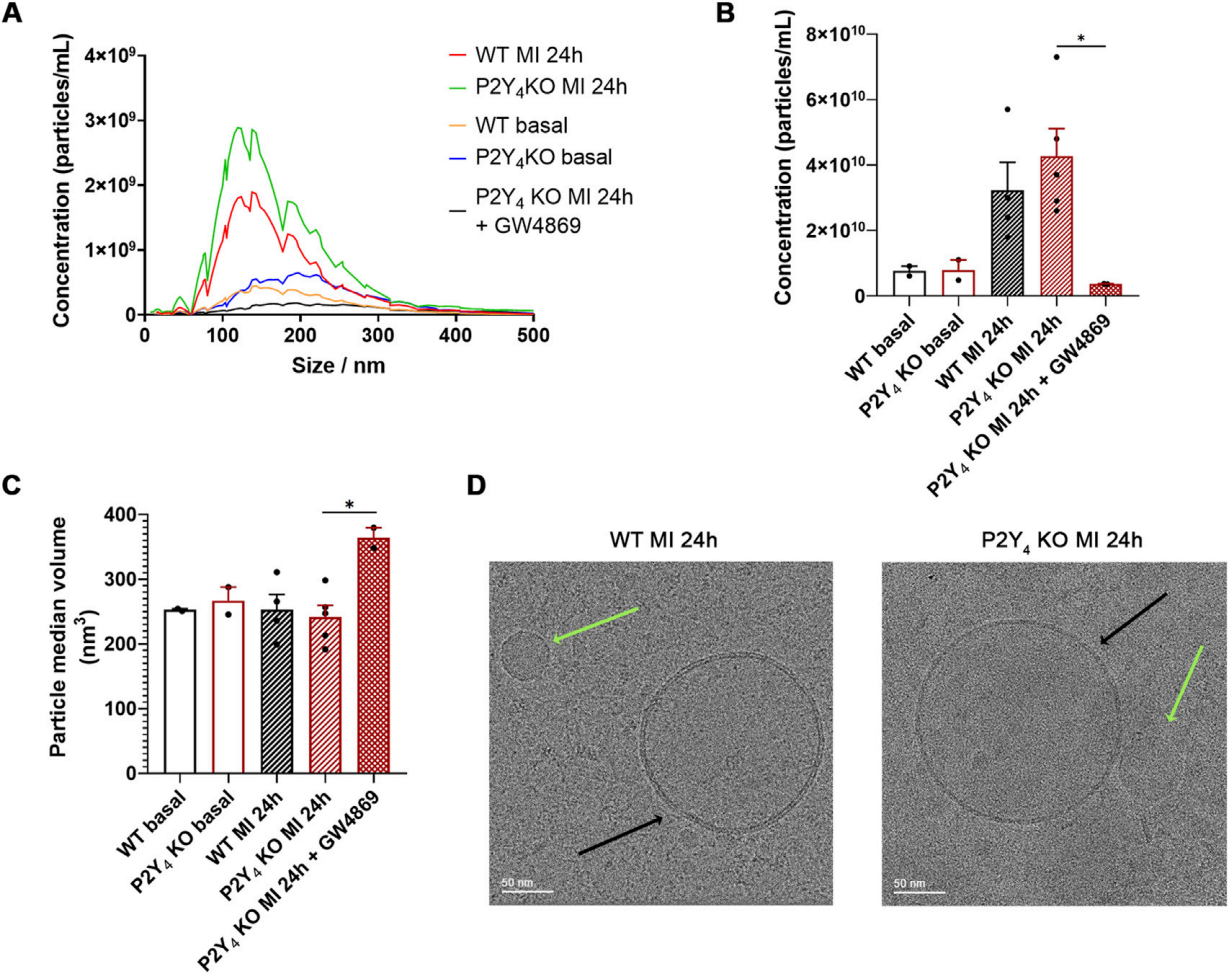
Nanoparticle tracking analysis (NTA) and transmission electron microscopy (TEM) analysis of plasma particles from WT and P2Y_4_ KO mice. **(A)** Size distribution calculated by tracking analysis of particles isolated using the Exo-spin Exosome Purification Kit from plasma of WT and P2Y_4_ KO mice, without MI (basal), 24 h post-MI (MI 24 h) or 24 h post-MI injected intraperitoneally with exosome inhibitor GW4869 (2.5 μg/g), 1 h before LAD ligation (MI 24 h + GW4869). **(B,C)** Concentration and median volume of particles isolated using the Exo-spin Exosome Purification Kit from plasma of WT and P2Y_4_ KO mice, without MI (basal), 24 h post-MI (MI 24 h) or 24 h post-MI injected intraperitoneally with exosome inhibitor GW4869 (2.5 μg/g), 1 h before LAD ligation (MI 24 h + GW4869). The size distribution, concentration and mean volume of plasma particles were determined using a Nanoparticle Tracking Analyzer (Zetaview). The data were processed using the in-build ZetaView Software (8.05.12 SP1). Data represent mean ± SEM. The sex ratio was 50% male and 50% female in each group (n = 2–4), except for the P2Y_4_ KO MI 24 h group (n = 5), which included 40% males and 60% females. **(D)** Cryo-transmission electron microscopy representative images of particles isolated using the Exo-spin Exosome Purification Kit from plasma of WT and P2Y_4_ KO mice, 24 h post-MI (MI 24 h). Black and green arrows indicate respectively detected exosomes and lipoproteins. Data were analyzed using a three-way ANOVA followed by a Bonferroni *post hoc* test. Data represent mean ± SEM. *p < 0.05.

We performed nanotracking particle analysis (NTA) and transmission electron microscopy (TEM) experiments to validate the efficacy of the used Exo-spin isolation kit on our plasma samples and to characterize further our exosomal preparations. NTA experiments were used to determine the concentration and median volume of particles isolated using the Exo-spin kit from plasma samples of WT and P2Y_4_ KO mice, without MI, 24 h post-MI or 24 h post-MI injected intraperitoneally with exosome inhibitor GW4869 (2.5 μg/g), 1 h before LAD ligation. The size distribution, concentration and median volume of plasmatic particles were determined using a Nanoparticle Tracking Analyzer (Zetaview) (Figures 4A–C).

Calculated particle sizes were compatible with the predominant presence of small-sized particles, exosomes, in our plasma particle preparations obtained using the Exo-spin kit (Figure 4A). We observed that particle concentration tend to increase more in P2Y_4_ KO mice than in WT mice, 24 h after LAD ligation (Figures 4A,B). Effectively, particle concentrations were respectively 4,2 ± 0,8.10^10^ particles/mL and 3,2 ± 0,9.10^10^ particles/mL in the plasma of P2Y_4_ KO mice and WT ischemic mice (mean ± SEM, p = 0,4) (Figure 4B). No significant difference in median volume was observed between plasma particles from P2Y_4_ KO and WT mice (Figure 4C). Median volume particle size in plasma preparations from P2Y_4_ KO and WT ischemic mice was, respectively 241,3 ± 18,4 nm^3^ and 252,7 ± 23,7 nm^3^ (mean ± SEM, p = 0,99) (Figure 4C). We have also investigated the effect of a single injection of the GW4869 exosome inhibitor, 1 h prior to LAD ligation in our NTA experiments. A drastic inhibition of particle production was confirmed in P2Y_4_ KO mice injected with GW4869 (2.5 μg/g) 1 h before LAD ligation (Figures 4A,B). Moreover the remaining detected particles in GW4869-injected mice were characterized by a higher mean volume (Figure 4C). Median volume particle size was respectively 241,3 ± 18,4 nm^3^ and 363,8 ± 15,7 nm^3^ in plasma preparations from P2Y_4_ KO mice and from GW4869-injected P2Y_4_ KO mice (mean ± SEM; *p < 0.05) (Figure 4C).

We have also performed Cryo-TEM experiments on particle preparations isolated using the Exo-spin Exosome Purification Kit from plasma of WT and P2Y_4_ KO mice, 24 h post-MI (Figure 4D). The presence of exosomes indicated by black arrows was clearly identified thanks to their lipid bilayer (Figure 4D). Other vesicles, most probably lipoproteins, were also detected in some fields and indicated by green arrows (Figure 4D). A deeper characterization would be needed to quantify and characterize the multiple subpopulations of exosomes of various sizes that were observed in both plasma preparations from WT and P2Y_4_ KO ischemic mice.

### Regulation of plasma exosome release in P2Y_4_ KO ischemic mice

**FIGURE 5:**
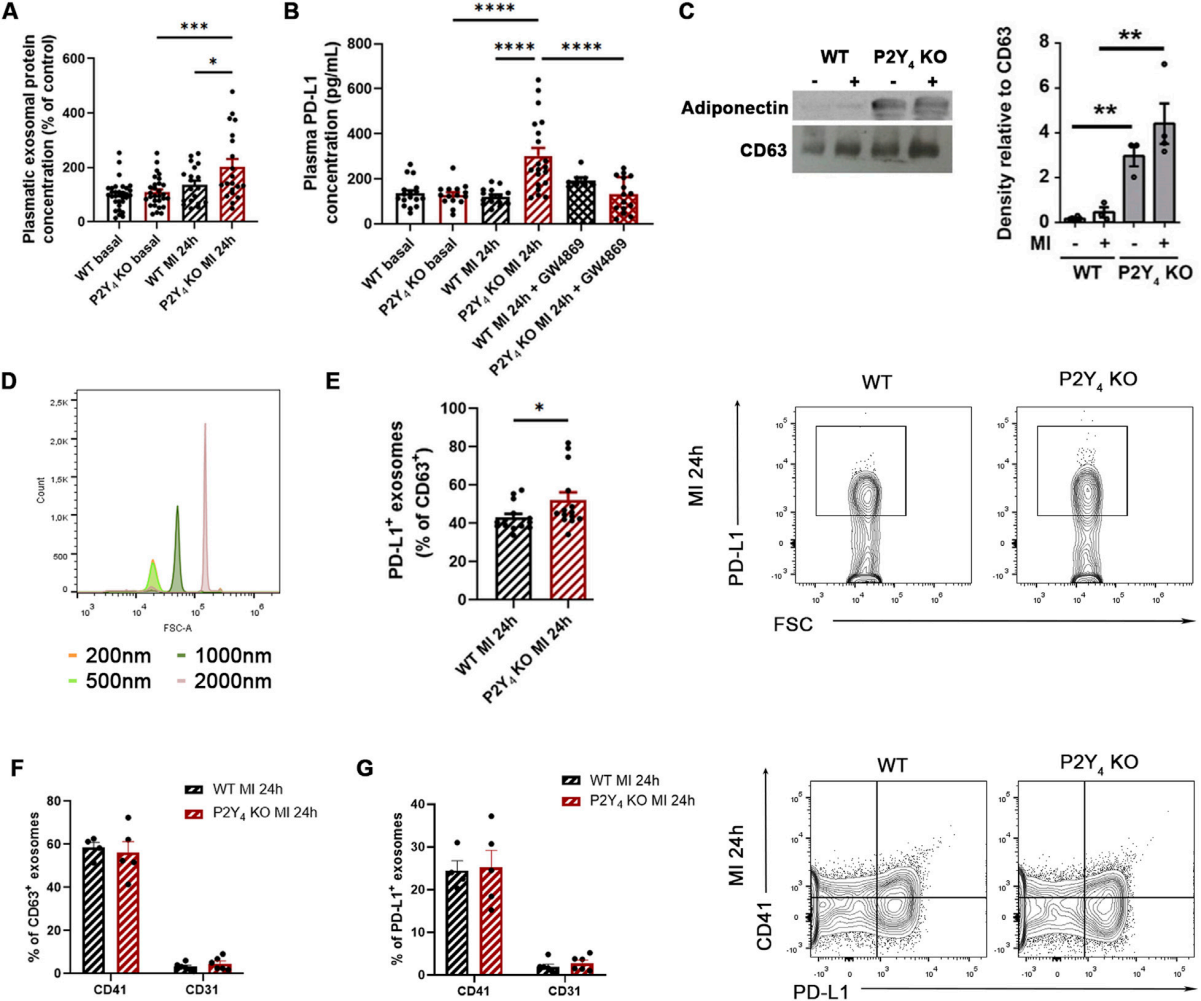
Increase in plasma PD-L1 concentration and PD-L1^+^ exosome number in the plasma of ischemic P2Y_4_ KO compared to WT mice. **(A)** Quantification of exosomal protein concentration by BCA assay on Exo-spin preparations from the plasma of WT and P2Y_4_ KO mice, without MI (basal) or 24 h after MI (MI 24 h) (n = 20). The sex ratio was 40% male and 60% female in each group (n = 30 for basal conditions, n = 20 for MI conditions). Data were analyzed using a two-way ANOVA followed by a Bonferroni *post hoc* test. **(B)** Quantification of PD-L1 plasma concentrations by ELISA in WT and P2Y_4_ KO mice, without MI (basal), 24 h post-MI (MI 24 h) or 24 h post-MI injected intraperitoneally with exosome inhibitor GW4869 (2.5 μg/g), 1 h before LAD ligation (MI 24 h + GW4869). The sex ratio was 50% male and 50% female in each group (n = 8–20), except for the P2Y_4_ KO basal, WT MI 24h, and P2Y_4_ KO MI 24 h + GW4869 groups (n = 15), which included 40% males and 60% females. Data were analyzed using a three-way ANOVA followed by a Bonferroni *post hoc* test. **(C)** Detection of adiponectin and CD63 exosome marker by Western blotting using exosomes from plasma of WT and P2Y_4_ KO mice, without MI or 24 h post-MI (left graph). Quantification of adiponectin band density, normalized to CD63 band density. The sex ratio was 50% male and 50% female in each group (n = 4) (right graph). Data were analyzed using a two-way ANOVA followed by a Bonferroni *post hoc* test. **(D)** Quantification of different sized beads (200 nm–2000 nm) by flow cytometry. **(E)** Quantification of PD-L1^+^ particles (expressed as percentage of CD63^+^ exosomes) by flow cytometry, isolated using the Exo-spin Exosome Purification kit on the plasma of WT and P2Y_4_ KO mice, 24 h after MI (MI 24 h). The sex ratio was 50% male and 50% female in both the WT MI 24 h and P2Y_4_ KO MI 24 h groups (n = 14). Data were analyzed using an unpaired, two-tailed Student’s t-test (left graph). Representative flow cytometry dotplots for PD-L1^+^ particles in the plasma of WT and P2Y_4_ KO mice, 24 h after MI (MI 24 h) (right graph). **(F)** Quantification of CD41^+^ and CD31^+^ particles (expressed as percentage of CD63^+^ exosomes) by flow cytometry, isolated using the Exo-spin Exosome Purification kit on the plasma of WT and P2Y_4_ KO mice, 24 h after MI (MI 24 h). The sex ratio was 50% male and 50% female in each group (n = 4–6), except for the CD41/CD63 staining in the P2Y_4_ KO MI 24 h group (n = 5), which included 40% males and 60% females. **(G)** Quantification of CD41^+^ and CD31^+^ particles (expressed as percentage of PD-L1^+^ exosomes) by flow cytometry, isolated using the Exo-spin Exosome Purification kit on the plasma of WT and P2Y_4_ KO mice, 24 h after MI (MI 24 h) (n = 4–6) The sex ratio was 50% male and 50% female in each group (n = 4–6), except for the CD41/PD-L1 staining in the P2Y_4_ KO MI 24 h group (n = 5), which included 40% males and 60% females (left graph). Representative flow cytometry dotplots for CD41/PD-L1 staining of particles in the plasma of WT and P2Y_4_ KO mice, 24 h after MI (MI 24 h) (right graph). Data represent mean ± SEM. *p < 0.05; **p < 0.01; ***p < 0.001; ****p < 0.0001.

To confirm our NTA data, we investigated if loss of the P2Y_4_ receptor was correlated with higher plasma exosome production. Using the Exo-spin isolation kit followed by BCA quantification, we found a higher level of exosomal protein content in the plasma of P2Y_4_ KO ischemic mice, compared with WT ischemic mice (231.1% ± 33.9% of control vs. 141.6% ± 18.5% of control, p = 0.0197) (Figure 5A). As shown in NTA experiments (Figures 4A,B), the level of plasma particles appeared to be higher in ischemic P2Y_4_ KO mice than in ischemic WT mice (Figure 5A).

We investigated then the potential regulation of the PD-L1 exosomal form in P2Y_4_ KO and WT ischemic mice. PD-L1 plasma level was quantified by ELISA after GW4869 exosome inhibitor injection, 24 h post-LAD. The higher concentration of PD-L1 observed in the plasma of P2Y_4_ KO ischemic mice compared to WT ischemic mice (302.03 ± 35.71 pg/mL vs. 121.03 ± 9.53 pg/mL, p < 0.0001) was no longer visible in P2Y_4_ KO mice intraperitoneally injected with GW4869 (2.5 μg/g), 1 h before LAD ligation (Figure 5B). The inhibitory effect of GW4869 supports that plasma PD-L1 overexpression detected by ELISA in P2Y_4_ KO ischemic mice represents an increase in its exosomal form (Figure 5B).

To investigate a potential increase of plasma adipose tissue-exosomes (AT-exosomes) in P2Y_4_ KO ischemic mice, Western blot analysis was chosen to detect adiponectin in combination with CD63 exosome marker in plasma protein extracts. We performed immunoblot analysis of CD63 exosome marker and adiponectin expression on Exo-spin preparations from our plasma of WT and P2Y_4_ KO basal and ischemic (MI 24 h) mice (Figure 5C). CD63 staining supports the higher presence of exosomes in our P2Y_4_ KO plasma exosome samples (Figure 5C), as shown by BCA assay (Figure 5A). The increase of plasma exosomal proteins in plasma samples of P2Y_4_ KO compared to WT ischemic mice appears thus to be related to an increased number of exosomes (Figures 5A,C). Interestingly, adiponectin staining is strongly detected in exosomes lysates from P2Y_4_ KO mice, with or without MI, and only barely detectable in exosome lysate from post-MI WT mice (Figure 5C). P2Y_4_ KO mice are thus characterized by an increase in exosomal adiponectin, possibly reflecting higher production of AT-exosomes in their plasma.

### Increase of PD-L1^+^ plasma exosomes in P2Y_4_ KO ischemic mice

The technical issues encountered using flow cytometry to characterize small particles such as exosomes can be reduced by improving scatter resolution and lowering background noise (van der Vlist et al., 2012; Robert et al., 2012). We have setup flow cytometry experiments using fluorescent beads with different sizes (200, 500, 1,000 and 2000 nm) (Flow Cytometry Sub-micron Particle Size Reference kit, Invitrogen™) for the gating on vesicles having a size range corresponding to exosomes. As shown in Figure 5D, it was possible to discriminate 500–2000 nm beads but flow cytometry experiments were not able to distinguish events corresponding to particles having a size below 500 nm. Plasma particle preparations were obtained from ischemic WT and P2Y_4_ KO mice, 24 h post-LAD, using the Exo-spin kit. We quantified the proportion of CD63^+^ PD-L1^+^ events, using antibodies against CD63 and PD-L1 (Figure 5E). We observed an increase of PD-L1^+^ plasma particles in P2Y_4_ KO mice compared to WT mice (51.94% ± 4.09% vs. 42.94% ± 1.92%, p = 0.028) (Figure 5E).

The increased level of adiponectin observed by Western blotting in plasma exosomes from P2Y_4_ KO mice supports an increase of exosome from adipocyte origin (Figure 5C). To explore additional potential sources of anti-inflammatory exosomes, we used the platelet marker CD41 and the endothelial marker CD31, either alone or in combination with PD-L1 (Figures 5F,G). Endothelial cells, like adipocytes, express high levels of CDH13, an adiponectin receptor involved in exosome release (De et al., 2010; Parker-Duffen et al., 2013). Platelet origin was investigated because they represent a major source of exosomes derived from circulating cells (Liu et al., 2021). We found a comparable level of CD41^+^ plasma exosomes for WT and P2Y_4_ KO ischemic mice, respectively 58.4% ± 2.5% vs. 56.0% ± 5.2% of CD63^+^ exosomes (mean ± SEM) (Figure 5F). No significant difference in CD41^+^/PD-L1^+^ plasma exosomes was detected between WT and P2Y_4_ KO ischemic mice, respectively 24.4% ± 2.3% vs. 25.2% ± 4.0% (mean ± SEM) (Figure 5G). We observed that plasma CD31^+^ exosomes represent a small proportion of our plasma exosomes, respectively 3.2% ± 0.7% vs. 4.7% ± 1.2% of CD63^+^ exosomes (mean ± SEM) (Figure 5F). No significant difference in plasma CD31^+^/PD-L1^+^ exosomes was observed between WT and P2Y_4_ KO ischemic mice, respectively 1.9% ± 0.6% vs. 2.8% ± 0.7% (mean ± SEM) (Figures 5F,G). Together, these data indicate that the exosome subpopulation increased in P2Y_4_ KO ischemic mice is not derived from endothelial cells or platelets. The elevated levels of exosomal adiponectin observed by Western blotting in plasma exosomes from P2Y_4_ KO mice (Figure 5C) support an adipocyte origin. Our attempts to use perilipin and UCP-1 as adipocyte markers in flow cytometry were not successful, as exosome permeabilization caused a loss of membrane integrity. Although flow cytometry has limited sensitivity for in-depth analysis of plasma exosome subpopulations, the adipose tissue origin of exosomes in P2Y_4_ KO mice could be further investigated using alternative and validated adipocyte-specific membrane markers.

### Plasma exosomes of ischemic P2Y_4_ KO mice can induce *in vitro* anti-inflammatory macrophage polarization

**FIGURE 6:**
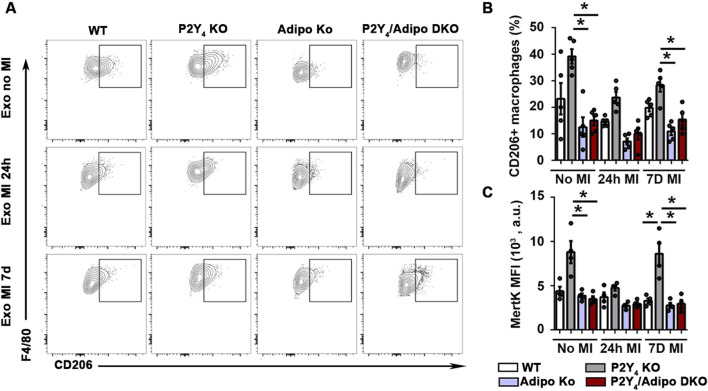
Plasma exosomes isolated from P2Y_4_ KO ischemic mice can induce *in vitro* macrophage polarization. **(A)** Flow cytometry representative dotplots of F4/80 and CD206 expression in bone marrow-derived macrophages stimulated with exosomes extracted from the plasma of WT, P2Y_4_ KO mice, adiponectin KO (AdipoKO) and P2Y_4_/adiponectin double KO (P2Y_4_/Adipo DKO) mice, 24 h (MI 24 h) or 7 days (MI 7d) after MI. **(B)** Quantification of CD206^+^ macrophages in bone marrow-derived macrophages (expressed as percentage), stimulated with exosomes extracted from WT, P2Y_4_ KO, Adipo KO and P2Y_4_/Adipo DKO mice without MI (basal), or 24 h (MI 24 h) or 7 days (MI 7d) post-MI. The sex ratio was 40% male and 60% female in each group (n = 5). **(C)** Mean fluorescence intensities (MFI, arbitrary units) of MerTK staining on bone marrow-derived macrophages, stimulated with exosomes extracted from WT,P2Y_4_ KO, Adipo KO and P2Y_4_/Adipo DKO mice without MI (basal), or 24 h (MI 24 h) or 7 days (MI 7d) post-MI. The sex ratio was 50% male and 50% female in each group (n = 4). Data were analyzed using a two-way ANOVA followed by a Bonferroni *post hoc* test. Data represent mean ± SEM. *p < 0.05.

To address the possible anti-inflammatory action of plasma exosomes released in ischemic P2Y_4_ KO mice, we compared the capacity of plasma exosomes isolated from ischemic WT, P2Y_4_ KO, adiponectin KO mice and P2Y_4_/adiponectin double KO mice to regulate macrophage polarization. Mouse WT bone marrow-derived macrophages were isolated and stimulated with plasma exosomes obtained from ischemic mice, 24h and 7 days after MI. A higher number of macrophages positive for F4/80 and CD206 macrophage activation marker was observed after stimulation with plasma exosomes from P2Y_4_ KO mice than with plasma exosomes from WT mice (Figures 6A,B). Additionally, plasma exosomes from P2Y_4_ KO mice increased significantly the number of CD206^+^ macrophages positive for MerTK, indicating a M2c anti-inflammatory polarization (Figure 6C). The capacity to induce M2c polarization was observed for plasma exosomes collected from non-ischemic P2Y_4_ KO mice and from ischemic P2Y_4_ KO mice, 7 days post-MI but not 24 h post-MI (Figure 6C). Interestingly, the ability to promote M2c polarization was not observed with exosomes derived from P2Y_4_/adiponectin double KO mice (Figure 6C).

## Discussion

### Exosome inhibitor injection suppresses cardioprotection observed in P2Y_4_ KO ischemic mice

Exosomes are now recognized as important actors in the regulation of many physiological and pathophysiological mechanisms, including the post-ischemic inflammatory response. The present study investigates the presence of anti-inflammatory exosomes in the plasma of P2Y_4_ KO mice and their contribution to the protection against MI observed in these mice. Reduced neutrophil infiltration and reduced expression of adhesion molecules (ICAM-1, VCAM-1, E-selectin), and metalloproteases (MMP-8, MMP-9) were previously reported in ischemic hearts of P2Y_4_ KO mice (Horckmans et al., 2015). In a first approach, we demonstrated the action of an intraperitoneal injection of the neutral N-SMase inhibitor, GW4869, on the ischemic heart and PAT of P2Y_4_ KO mice. Analysis of ischemic heart and PAT was performed 7 days after LAD ligation in P2Y_4_ KO mice injected with exosome inhibitor GW4869 during MI onset. Interestingly, GW4869 injection abolished the reduction of cardiac fibrosis area and T cell infiltration observed in their heart, compared to WT mice, 7 days post-MI. Besides these effects in the heart, we investigated a potential action of GW4869 injection in the PAT of P2Y_4_ KO ischemic mice. We previously reported a reduced post-ischemic expansion of fat-associated lymphoid clusters in these mice, correlated with a higher level of anti-inflammatory Treg lymphocytes and M2c macrophages (Horckmans et al., 2022a). We observed here that GW4869 injection totally inhibited the increase of T cell apoptosis in the ischemic P2Y_4_ KO PAT. The data obtained with GW4869 exosome inhibitor support that the reduction of cardiac inflammation induced by P2Y_4_ receptor loss in the ischemic heart and PAT, is exosome-dependent. Of course the plasma of ischemic mice contains various pro- and anti-inflammatory exosomes released after LAD ligation by circulating cells such as leukocytes and platelets, inflamed adipose tissues and the ischemic heart (Yuan et al., 2016). Nevertheless the GW4869-induced pro-inflammatory action in P2Y_4_ KO mice supports the hypothesis of an increased amount of anti-inflammatory exosomes in these mice compared to WT mice after ischemia.

GW4869 blocks exosome biogenesis through an ESCRT (Endosomal Sorting Complexes Required for Transport)-independent pathway. We considered other exosome inhibitors such as manumycin A which targets ESCRT-dependent exosome release. However, to our knowledge there are no reported *in vivo* studies using manumycin A administration. Establishing safe and effective doses for exosome inhibition would be essential before considering its use in future *in vivo* studies.

### P2Y_4_ receptor loss regulates adiponectin/CDH13 system involved in exosome biogenesis

P2Y_4_ receptor expression has been demonstrated in cardiac endothelial cells, adipocytes and adipose-derived stem cells (Lemaire et al., 2017; Horckmans et al., 2022a). P2Y_4_ loss induces increased adiponectin secretion by cardiac adipocytes (Lemaire et al., 2017). Recent studies have shown that adiponectin produced from adipocytes accumulates in tissues such as heart and skeletal muscles through interaction with the CDH13 adiponectin receptor, also named T-cadherin (De et al., 2010; Parker-Duffen et al., 2013; Matsuda et al., 2015). Obata et al. demonstrated that the adiponectin/CDH13 system enhances exosome biogenesis and secretion by endothelial cells (Obata et al., 2018). Our previous analysis of the inflammatory state of the PAT in P2Y_4_ KO mice has shown a higher level of anti-inflammatory M2c macrophages and regulatory T cells, correlated with reduced post-ischemic expansion of fat-associated lymphoid clusters (Horckmans et al., 2022a). We also demonstrated that P2Y_4_ KO mice displayed adipocyte beiging with increased PD-L1 expression in their PAT after LAD ligation, compared to WT mice (Horckmans et al., 2022a). Interestingly, we observed here the overexpression of both adiponectin and CDH13 in the PAT of P2Y_4_ KO mice. Loss of P2Y_4_ receptor could thus be correlated with a local adiponectin-dependent exosome release regulating lymphocyte infiltration in the ischemic PAT and heart. Additionally loss of P2Y_4_ in other inflamed adipose tissues could explain the observed increase of plasma AT-exosomes in P2Y_4_ KO ischemic mice. Adiponectin is known to have an anti-inflammatory action and to stimulate the secretion of exosomes by various cell and tissue types, including endothelial cells (Obata et al., 2018), renal pericytes (Tsugawa-Shimizu et al., 2021), MSCs (Nakamura et al., 2020), muscle (Tanaka et al., 2019), aorta (Kita et al., 2019) and heart (Kita and Shimomura, 2021). Adiponectin can induce exosome secretion by CDH13–expressing cells in the heart and coronary arteries and can influence plasma levels of exosomes which may have cardioprotective effects (Kita et al., 2019; Kita and Shimomura, 2021). CDH13 is expressed on endothelial cells, platelets, leukocytes and also on adipocytes (Goddeke et al., 2018). Interestingly CDH13 abundance differs according to metabolic disorders and is decreased in obese mouse models (Goddeke et al., 2018). Its role in adipose tissue is not yet elucidated: it can interfere with adipocyte differentiation potential and might reflect the health status of adipose tissue (Goddeke et al., 2018). Our data point a possible role of adipose CDH13 in the production of cardioprotective exosomes. Overexpression of adiponectin in P2Y_4_ KO mice could stimulate additional exosome secretion in a variety of tissues and cells expressing CDH13. Flow cytometry revealed comparable number of plasma exosomes derived from platelets or endothelial cells in P2Y_4_ KO and WT ischemic mice. The increased presence of plasma exosomes containing adiponectin in P2Y_4_ KO ischemic mice supports their adipose tissue origin. Adiponectin is a circulating adipokine produced by adipocytes but is also found within exosomes (Obata et al., 2018). Adiponectin could thus stimulate the release of its own exosomal form by CDH13-expressing adipocytes and induce the secretion of exosomes by other CDH13-expressing cells. Of course the expression of the P2Y_4_ receptor in circulating cell populations and specific vessels and other adipose tissues in ischemic conditions has to be further investigated. Technical progress has to be made worldwide in the characterization of exosome subsets to elucidate precisely the various potential origins of plasma exosomes. Although flow cytometry has limited sensitivity for in-depth analysis of plasma exosome subpopulations, the adipose tissue origin of exosomes in P2Y_4_ KO mice, supported by our exosomal adiponectin data, could be further investigated using validated adipocyte-specific membrane markers. More generally, we are aware that the use of a constitutive global KO has certain limitations to clearly define the source of anti-inflammatory exosomes increased in P2Y_4_ KO mice. The present study could be expanded once conditional KO or adipose tissue-specific KO models for the P2Y_4_ receptor become available.

### P2Y_4_ receptor loss induces increased post-MI plasma expression of exosomal PD-L1

We demonstrated here that cardioprotection was lost in P2Y_4_ KO mice after the intraperitoneal injection of GW4869 exosome inhibitor, as previously observed using an anti-PD-L1 blocking antibody (Horckmans et al., 2022a). The inhibitory effect of GW4869 on plasma PD-L1 increase in P2Y_4_ KO ischemic mice shown by ELISA as well as the higher detection of PD-L1^+^ exosomes by flow cytometry support higher expression of plasma PD-L1 exosomal form in these mice. Exosomal PD-L1 is an important actor of immunosuppression and considered as a predictor for anti-PD-1 therapy (Chen et al., 2018). Tumor cells can evade immune surveillance through PD-L1, interacting with the PD-1 receptor on T cells and also through PD-L1 secretion in tumor-derived exosomes. (Poggio et al.). We have shown previously an increase of Tregs and beige adipocytes in the PAT of P2Y_4_ KO mice (Horckmans et al., 2022a). The role of the PD-1/PD-L1 axis in Treg function has already been demonstrated (Cai et al., 2019). Adipose tissue has already been described as a reservoir of Foxp3^+^ Treg cells (Zeng et al., 2018). They are abundant in the visceral adipose tissue of normal diet mice and their number is greatly reduced in insulin-resistant animal models of obesity (Zeng et al., 2018). This reduction in Treg leads to a pro-inflammatory state of visceral adipose tissue state and metabolic dysregulation in obese mice (Zeng et al., 2018). In case of obesity-associated sustained inflammation, white adipose tissue dysfunction leads to impaired secretion of adipokines, such as adiponectin and can be balanced by the activation of adipocyte browning (Hoerter et al., 2004; Waldén et al., 2012). In obese individuals, AT-exosomes are known to contribute to the development of insulin resistance via activation of adipose-resident macrophages and secretion of pro-inflammatory cytokines (Lei et al., 2021). A potential therapeutic approach to treat human obesity/diabetes would aim to increase the number of these beige adipocytes. PD-L1 is expressed in antigen-presenting cells, endothelial cells, platelets, leukocytes (Sharpe et al., 2007; Dermani et al., 2019; Eppihimer et al., 2002; Veluswamy et al., 2020) and is also a marker of beige adipocytes (Ingram et al., 2017). Besides its well-known role in immunosuppression in oncology, exosomal PD-L1 is known to play a role in skin wound (Su et al., 2019) and fracture healing (Lin et al., 2022) by binding to T cells expressing PD-1 and promoting tissue repair. The role of exosomal PD-L1 in the regulation of inflammation after MI was not yet investigated. Loss of cardioprotection in P2Y_4_ KO mice was previously observed after anti-PD-L1 blocking antibody injection (Horckmans et al., 2022a). The present study supports a potential anti-inflammatory role of exosomal PD-L1 and adiponectin contributing to protection against MI.

### Macrophage-polarization ability of plasma exosomes from P2Y_4_ KO mice

Even though we observed an increase of PD-L1^+^ plasma exosomes in P2Y_4_ KO mice, the potential anti-inflammatory action of their total plasma exosomes was important to investigate. The present study has evaluated the effect of total plasma exosomes isolated from P2Y_4_ KO and WT ischemic mice, 24h and 7 days post-MI, on bone-marrow derived macrophages. Interestingly we demonstrated the capacity of these exosomes to polarize macrophages into the M2c phenotype. We previously demonstrated the increase of anti-inflammatory M2c macrophage populations in the PAT of P2Y_4_ KO mice (Horckmans et al., 2022a). Anti-inflammatory exosomes released in the plasma of P2Y_4_ KO mice could contribute to increase macrophage polarization, 7 days post-MI, when M2 macrophages are needed to improve cardiac tissue repair.

All the altered regulatory mechanisms resulting from a gene deficiency and leading to an anti-inflammatory signal delivery are not easy to be determined. P2Y_4_ receptors expressed in endothelial cells and adipocytes can regulate the release of inflammatory mediators from inflamed heart and adipose tissues, and in the circulation. Even if the multiple sources and proportions of pro- and anti-inflammatory exosome subsets contained in the whole plasma have to be further characterized, the capacity of total plasma exosomes from P2Y_4_ KO ischemic mice to induce M2c-type polarization of macrophages *in vitro* is promising. Effectively, M2c macrophages are involved in the clearance of necrotic cells and have various pro-regenerative functions. After the acute inflammatory phase, reparative M2 macrophages are known to facilitate wound healing and cardiac regeneration by promoting fibroblast differentiation into myofibroblasts, collagen deposition as well as angiogenesis. M2c macrophages are characterized by MerTK expression, a tyrosine kinase which enables M2c macrophages to clear early apoptotic cells more efficiently than other macrophage subsets (Zizzo et al., 2012), and which is a negative regulator of T cell activation (Cabezón et al., 2015). MerTK plays a role in the regulation of immune checkpoint signaling through PD-L1/CD274 and PD-L2, which bind the programmed cell death 1 (PD-1) receptor on T cells, inhibiting their activation and promoting their apoptosis (Dong et al., 2002; Kasikara et al., 2017). PD-L1 is known to induce M2 macrophage polarization *in vitro* (Dong et al., 2002) and to promote a M2 phenotype in tumor-associated macrophages (Wei et al., 2021; Zhang et al., 2017; Zhu et al., 2020). Interestingly, exosomal PD-L1 was shown to facilitate M2 macrophage polarization (Yuan et al., 2022). M2c macrophages can also induce Foxp3 expression in human CD4 T cells having immunosuppressive activity (Schmidt et al., 2016).

NTA experiments have shown that MI induced an increase of plasma exosomes in both WT and P2Y_4_ KO mice, 24 h post-MI. BCA quantification assays and NTA showed that the level of plasma exosomes appears to be higher in P2Y_4_ KO than in WT ischemic mice. NTA experiments confirmed the inhibitory effect of a single injection of GW4869 on plasma exosome number, 1 h prior to LAD ligation. Effectively, a strong reduction of detected particles was observed and the remaining particles were characterized by a higher median volume. NTA and TEM experiments realized on our plasma exosome preparations confirmed the presence of exosome particles of various sizes in our plasma preparations. Even if exosomes were easily identified thanks to their lipid bilayer, other smaller vesicles that could be lipoproteins were also detected. The presence of lipoproteins has previously been identified in fresh plasma (Yuana et al., 2013) and could interfere in *vivo* experiments using plasma exosome preparations. Even if NTA experiments have not detected an important contamination by small vesicles, a deeper characterization and purification of exosome subpopulations will be needed in the future to plan the isolation of a sufficient amount of PD-L1^+^ exosomes from plasma particle preparations. Nevertheless it was very interesting to observe that the whole mixture of plasma exosome populations isolated from P2Y_4_ KO mice was able to induce M2c polarization *in vitro*. This ability was not observed for plasma exosomes isolated from P2Y_4_/adiponectin double KO mice.

We previously demonstrated the central role of PD-L1 in the cardioprotection resulting from P2Y_4_ receptor loss using a blocking anti-PD-L1 antibody (Horckmans et al., 2022a). Reduction of T cell infiltration in ischemic heart and increased T cell apoptosis in PAT were no more observed in P2Y_4_ KO mice after injection with an anti-PD-L1 blocking antibody (Horckmans et al., 2022a). The present study demonstrated the increase of plasma exosomal PD-L1 in P2Y_4_ KO ischemic mice and the negative effect of exosome inhibitor GW4869 injection on the cardioprotection observed in these mice. The quantification of the exosomal form of PD-L1 in MI patients could have a therapeutic interest and a prognostic value. More generally, the characterization and isolation of the various post-MI plasma exosome subsets would represent a very promising tool with powerful therapeutic applications. Nevertheless it constitutes a real challenge due to the size of these vesicular particles and the complexity of their multiple potential origins. Adipocytes are crucial regulators of the microenvironment, driving leukocyte polarization and regulating the adipose tissue inflammatory state. An overexpression of adiponectin and CDH13 could result in the release of anti-inflammatory exosomes from the PAT acting directly on the ischemic heart. An increase in PD-L1^+^ plasma exosomes could contribute to improve cardiac healing and outcome after MI by their anti-inflammatory action such as macrophage M2c polarization.

Mechanistic studies were focused on the ability of plasma exosomes to induce macrophage polarization which plays a central role in the resolution of cardiac inflammation. MerTK, the used marker of M2c macrophages, is also a negative regulator of T cell activation (Cabezón et al., 2015) and immune checkpoint signaling through PD-L1 (Dong et al., 2002; Kasikara et al., 2017). These experiments support that adiponectin overexpression in P2Y_4_ KO mice is essential for generating plasma exosomes capable of inducing M2c polarization. Adiponectin is known to regulate macrophage function and polarization by inducing an M2 polarization and stimulating production of anti-inflammatory IL10 (Mandal et al., 2011). Adiponectin overexpression could promote the M2 polarization of macrophages in the PAT. These anti-inflammatory macrophages could then be recruited into the ischemic myocardium after MI, where they can regulate inflammation, clear dead cells and debris and promote cardiac healing and repair. We previously showed an early reduction of inflammation in the ischemic PAT of P2Y_4_ KO mice, characterized by increased M2c and regulatory T (Treg) cell populations, decreased M1 macrophages and effector memory (Tem) CD4^+^ lymphocytes, and reduced fat-associated lymphoid cluster (FALCs) size compared with WT ischemic mice (Horckmans et al., 2022a). Our data support a mechanism of T cell inactivation/apoptosis occurring primarily in the PAT. The close PAT–heart interactions could explain the reduced myocardial T cell infiltration observed in P2Y_4_ KO mice in the context of increased PAT T cell apoptosis. Additional anti-inflammatory effects may also arise from the migration of exosome-induced M2c macrophages or anti-inflammatory exosomes released by the PAT, into the myocardium. Interestingly, T cell apoptosis was also increased in P2Y_4_ KO PAT compared with WT PAT in basal conditions. We previously showed that M2c macrophages counts were higher in the PAT of P2Y_4_ KO mice compared with WT mice at baseline (Horckmans et al., 2022a). This altered baseline inflammatory state of P2Y_4_ KO PAT could also contribute to the cardioprotective effects observed after LAD ligation.

## General conclusions

The identification and the regulation of post-MI beneficial exosomes that can shift macrophages progressively from a pro-inflammatory M1 phenotype to an anti-inflammatory M2 phenotype is very promising. The protective effects observed in P2Y_4_ KO mice are not solely due to an overall increase in exosome abundance, but rather to the balance between pro- and anti-inflammatory plasma exosomes and to specific cargo changes, particularly exosomal adiponectin and PD-L1.

Synergy between immune checkpoint targeting and exosome depletion using GW4869 has been demonstrated in anti-tumor therapy (Wang J. S. et al., 2025), but cardiotoxicity has been associated to PD-L1 inhibitors in cancer patients (Liu et al., 2022). CDH13 expression is linked to immune cell infiltration, affects cancer prognosis and can be downregulated by anti-PD1/CTLA-4/PD-L1 immunotherapy (Situ et al., 2024). The present study brings thus important information about the role of PD-L1 in non-immune processes and could help to understand the reported cardiac side effects of anti-PD-L1 treatments.

Our data support that among various exosome subsets, anti-inflammatory exosomes are released in the blood of P2Y_4_ KO ischemic mice in sufficient amounts to polarize macrophages *in vitro*. These plasma exosomes can regulate post-ischemic cardiac inflammation and repair, and thus post-MI outcome. Anti-inflammatory exosomes could operate during the post-MI inflammatory response and limit the extension of cardiac fibrosis. Exosomes are involved in many cardiovascular processes and have thus a great therapeutic potential: they are non-immunogenic, biocompatible and more stable than cells, resistant to cryo-conservation and constitute thus a major novel alternative to whole-cell therapies (Bellin et al., 2019). Therapeutic use of stem cell-derived exosomes has been described to regulate cardiac fibrosis, remodeling and angiogenesis after MI (Gallet et al., 2017).

We identified previously a polymorphism in human *P2RY*_*4*_ gene leading to a loss of function of the corresponding mutant N178T P2Y_4_ receptor (Horckmans et al., 2022b). This missense variant is largely present worldwide in the general population, more in healthy individuals than patients with coronary artery disease, and its presence in these patients is correlated with reduced cardiac severity and risk scores (Horckmans et al., 2022b). The study of plasma exosomes and exosomal PD-L1 level in patients carrying this specific N178T P2Y_4_ polymorphism and their potential link with the severity of infarction could have promising therapeutic applications. Exosomes play a determinant role in the complex immune and inflammatory response under cardiac ischemia. The targeting of specific exosome subsets in an anti-inflammatory approach could have a major therapeutic interest against MI. The present study demonstrates the presence of anti-inflammatory exosomes in a model of cardioprotection related to a nucleotide receptor loss, and could contribute to reinforce the efficacy of anti-inflammatory therapies to improve MI outcome.

## Data Availability

The original contributions presented in the study are included in the article/supplementary material, further inquiries can be directed to the corresponding author.
